# Mitochondrial Morphology and Function of the Pancreatic β-Cells INS-1 Model upon Chronic Exposure to Sub-Lethal Cadmium Doses

**DOI:** 10.3390/toxics6020020

**Published:** 2018-03-22

**Authors:** Adeline Jacquet, Cécile Cottet-Rousselle, Josiane Arnaud, Kevin Julien Saint Amand, Raoua Ben Messaoud, Marine Lénon, Christine Demeilliers, Jean-Marc Moulis

**Affiliations:** 1 Laboratory of Fundamental and Applied Bioenergetics (LBFA), Inserm, Universite Grenoble Alpes, 38000 Grenoble, France; adeline.jacquet1@orange.fr (A.J.); cecile.cottet@univ-grenoble-alpes.fr (C.C.-R.); jarnaud@chu-grenoble.fr (J.A.); jsakevin@gmail.com (K.J.S.A.); raouabenmessaoud@yahoo.com (R.B.M.); marine.lenon@hotmail.fr (M.L.); christine.demeilliers@univ-grenoble-alpes.fr (C.D.); 2 Biochemistry, Molecular Biology and Environmental Toxicology (SB2TE), Grenoble University Hospital, CS 10217, 38043 Grenoble, France; 3 CEA-Grenoble, Bioscience and Biotechnology Institute (BIG), 38054 Grenoble, France

**Keywords:** mitochondrial network, image analysis, mitochondrial morphology, bioenergetics, sub-lethal exposure, toxicological mechanism, cadmium

## Abstract

The impact of chronic cadmium exposure and slow accumulation on the occurrence and development of diabetes is controversial for human populations. Islets of Langerhans play a prominent role in the etiology of the disease, including by their ability to secrete insulin. Conversion of glucose increase into insulin secretion involves mitochondria. A rat model of pancreatic β-cells was exposed to largely sub-lethal levels of cadmium cations applied for the longest possible time. Cadmium entered cells at concentrations far below those inducing cell death and accumulated by factors reaching several hundred folds the basal level. The mitochondria reorganized in response to the challenge by favoring fission as measured by increased circularity at cadmium levels already ten-fold below the median lethal dose. However, the energy charge and respiratory flux devoted to adenosine triphosphate synthesis were only affected at the onset of cellular death. The present data indicate that mitochondria participate in the adaptation of β-cells to even a moderate cadmium burden without losing functionality, but their impairment in the long run may contribute to cellular dysfunction, when viability and β-cells mass are affected as observed in diabetes.

## 1. Introduction

As a widespread contaminant found in the environment, the metal cadmium and its toxicity remain the subject of continuous studies with different approaches. Indeed, low background cadmium contamination in soils, usually below 0.5 ppm, is contributed by dispersion from various sources, such as mining and refining of other metals, waste disposal, and the dispersal of phosphate fertilizers. Increased environmental impact occurs at some particular sites, such as smelters and waste incinerators [1]. Cadmium dispersion contaminates crops with cadmium passing through the food chain up to farming animals and human populations. Hence, food is a considerable source of cadmium exposure for humans. Recommendations have been issued and continue to be updated by regulatory bodies to minimize the health effect of cadmium contamination of food and drinking water [2,3]. But a major difficulty of the topic is that biological targets of cadmium are numerous [4], and the mechanisms of action of the Cd^2+^ cation, the only ionic state of biological relevance, can be complex [5,6]. Biochemically, this is mainly due to the electropolarity and the size of Cd^2+^ which enable a range of molecular interactions [6,7].

In animals and at the organ level, kidneys, liver, and the skeleton are well-established sites of deposition with cadmium-induced damage or functional impairment [8]. But other organs may also be affected by cadmium exposure [9] and the functional consequences are often difficult to delineate, particularly at low levels of exposure. A case in point is pancreas. It is a site where cadmium can be found [10], in the endocrine compartment, and even in populations without particularly high exposure [11]. Thus, the causative link between cadmium exposure and prevalence of type II diabetes is difficult to establish for human populations [12,13,14]. As an alternative to the screening of human populations, animal studies may bring illuminating information forth, but too many of them implement unduly high cadmium doses and inappropriate modes of exposure that do not help clarifying the debate [15].

Yet, for the above recalled biochemical reasons, cadmium may well be interfering with the function of the endocrine pancreas, and with insulin production in particular. The later process is the specialized function of β-cells enclosed in islets of Langerhans in response to circulating glucose concentrations. The general model (e.g., [16]) leads from glucose uptake to secretion of insulin granules with increased glycolysis and adenosine triphosphate (ATP) production, closure of potassium channels at the plasma membrane, calcium uptake, and stimulated exocytosis. Most, if not all, steps of this complex mechanism may be sensitive to the presence of cadmium, but which is/are the most susceptible remains unclear. This questioning is particularly worth considering when interest is focused on low levels of exposure [8,15,17], as is the most prevalent situation for human populations nowadays and for which mechanistic insight is expected to provide means for educated intervention modalities.

Previous work has targeted mitochondria as key organelles converting chemical (glucose) into electrical (membrane depolarization) signals via ATP in β-cells [16]. This segment lies in the upper region of the insulin secretion cellular process, and it is thus expected to have downstream consequences if deficient. Short term (24 h) and relatively high cadmium concentration exposure (above the median lethal dose at more than 5 μM) induced apoptosis of the β-cells rodent model RIN-m5F with dysfunction of mitochondria [18] as could be predicted [19,20]. However, the specific impact of doses of cadmium largely below the onset of cell death on the mitochondria of β-cells has never been reported in details. The present work is an attempt to make up for this gap by exploring the morphological and functional changes occurring to mitochondria of INS-1 cells as a function of the longest possible time of exposure to largely sub-lethal concentrations of the Cd^2+^ cation. We tried to separate cellular events occurring either largely before or upon cell death. It thus appeared that mitochondria do sense moderate levels of cadmium, but cells can cope with such a challenge, and functional consequences are only observed when cells begin to die.

## 2. Materials and Methods

### 2.1. Cells and Treatments

INS-1 is a pancreatic β-cell line which was isolated from X-ray induced rat insulinoma [21]. It was obtained from the Department of Genetic Medicine and Development, University of Geneva Medical Center (Switzerland). They were maintained in RPMI 1640 medium containing 2 g/L glucose, supplemented with 10% foetal bovine serum (FBS), antibiotics (1% of penicillin (10,000 U/mL) and streptomycin (10 mg/mL)), 50 μM 2 mercaptoethanol, 1 mM sodium pyruvate and 2 mM Glutamine (complete medium) and grown in a humidified incubator with 5% CO_2_ at 37 °C.

A CdCl_2_ solution (250 μM) in PBS was added to the medium at appropriate volumes to achieve the intended end concentrations after cell adhesion. Seeding was at 1.5 × 10^5^ cells/mL and growth occurred either in complete medium (control) or in complete medium containing the required concentration of CdCl_2_. Incubation was for 72 or 96 h with regular medium replacement at 37 °C, 5% CO_2_.

### 2.2. Viability Measurements

To assess viability, INS-1 cells were exposed to CdCl_2_ in the complete culture medium at different concentrations in the [0–20] μM range for 72 h. At the end of the treatment, cells were washed with PBS and brought in suspension by incubation with 0.25% trypsin/EDTA in PBS without calcium and magnesium. Cells were pelleted at 150× *g* for 5 min, the pellet was rinsed with PBS, and suspended at 10^6^ cells/mL in 50 mM HEPES, 0.7 M NaCl, 12.5 mM CaCl_2_, pH 7.4. The suspension was labeled with Fluoprobe 488-annexin V (Interchim) then 1 μg/mL propidium iodide (PI) for 15 min at room temperature in the dark. The stained cells were detected by flow cytometry with a LSR Fortessa™ cell analyzer (Becton Dickinson, Le Pont de Claix, France) using the 488 nm sapphire laser and 532 nm compass laser for Fluoprobe 488 and PI, respectively. The corresponding fluorescence emission was measured with a 525/50 nm and 585/15 nm band-pass filters, respectively. Live cells are not labeled in this assay, whereas preapoptotic ones bind annexin V, necrotic ones accumulate PI, and doubly labeled cells are the dead ones. As an alternative method to the above labeling of cells, viability was also measured with the Cell Titer 96^®^ AQ_ueous_ One Solution Cell Proliferation Assay (Promega, Madison, WI, USA) in 96 well plates until adherence, then cadmium was added at different concentrations as explained above. The number of cells able to reduce the MTS tetrazolium compound was determined by recording the absorbance at 490 nm with a multi-well plate reader (Clariostar, BMG Labtech, Ortenberg, Germany).

### 2.3. Immunofluorescence Measurements

In immunofluorescence (IF) experiments, INS-1 cells were inoculated at 5000 cells/well on culture slides with detachable culture chambers (Falcon/Corning) until adherence. They were treated with different concentrations of CdCl_2_ for 96 h as described above. In wells in which mitochondria were labeled without nuclear staining, the cell-permeable fluorescent probe MitoTracker Red CMXRos (ThermoFisher, Illkirch, France) was added at 200 nM for 30 min at 37 °C. Cells were fixed in fresh 4% paraformaldehyde for 10 min at ambient temperature, washed twice with PBS, then cells were permeabilized using 0.2% Triton X-100 in PBS for 15 min, rinsed thrice, and blocked with PBS-Tween (1 mg/mL) BSA 5% (PBS-T BSA) for 1 h at 37 °C. Mitochondria were alternatively labeled with the primary antibody (D6D9 Rabbit mAb, Cell Signaling Technology, Danvers, MA, USA) raised in rabbit against mitochondrial aconitase (the product of the ACO_2_ gene) as an alternative to MitoTracker staining. The mAb was diluted 200 fold in PBS-T BSA and cells were incubated overnight at 4 °C. The cells were then rinsed thrice with PBS, and the primary antibody was reacted for 4 h at room temperature in the dark with the labeled secondary one (goat anti rabbit secondary antibody Hylite Fluor^®^ 488, Anaspec–Eurogentec, Angers, France) diluted 200 fold in PBS-T BSA. Before the end of the latter incubation, nuclear staining was performed with PI (1 mg/mL) for 20 min at 4 °C. Culture chambers were removed, and slides were mounted and sealed before microscopic observation.

A Leica TCS SP8 inverted laser scanning confocal microscope (Leica Microsystems, Wetzlar, Germany) equipped with a 40× Oil immersion objective was used to collect images. Laser excitation was 488 nm for Hylite Fluor 488, 552 nm for MitoTracker Red CMXRos and PI, with fluorescence emission at 500–550 nm, 575–630 nm, and 605–685 nm, respectively. The Mitotracker probe was used to cross check the images recorded by labeling aconitase: both sets of images qualitatively agreed and, since the latter were of better quality than the former, only wells in which aconitase was detected were analyzed in details. Several fields were recorded for each slide and quantitative analysis with the Image J (imagej.nih.gov) and Volocity (Improvision, Perkin-Elmer, Courtaboeuf, France) computer programs was carried out on all of them as follows. In a first step, tophat filtering was applied to the images recorded with the mitochondrial channel (aconitase fluorescence) in Image J to remove noise and to obtain a precise definition of the mitochondrial morphology. The filtered images were then analyzed with the Volocity software which provides morphological parameters like perimeter, area, skeletal length and diameter for each identified object. Each analyzed Cd-treatment group corresponded to tens of cells, and hundreds or thousands of mitochondrial objects. From these data, the circularity shape factor was calculated, as Equation (1).

circularity shape factor = 4π × mean area/(mean perimeter)^2^ (1)

### 2.4. ATP Measurements

To measure the cellular content in adenosine nucleotides, cells were rinsed with PBS after the cadmium treatment as described above. Cellular perchlorate extracts were prepared with 750 μL of cold 2.5% (*w*:*v*) HClO_4_-EDTA 6.25 mM. The recovered mixture was strongly mixed and centrifuged at 12,000× *g* for 5 min at 4 °C. The supernatant was neutralized at pH 7 with MOPS-KOH buffer 0.3 M and centrifuged 2 min at 12,000× *g*. Aliquots (75 μL) of the supernatant were then mixed with 15 μL of HCl 1 M and 35 μL of 28 mM pyrophosphate buffer pH 5.75. Thirty µl of the mixture were analyzed on a Polaris 5 C18-A, Agilent S (250 × 4.6 mm) column equilibrated in pyrophosphate buffer pH 5.75 at 1 mL/min. The retention times of ATP, adenosine diphosphate (ADP) and adenosine monophosphate (AMP) are approximately 6, 7 and 12 min, respectively, as determined by UV absorption at 254 nm.

### 2.5. Respiration Rates

The oxygen consumption rates of INS-1 cells were measured with a temperature-controlled Hansatech oxygraph equipped with a Clark electrode and monitored with the *oxygraph* software. For measurements with glucose stimulation, harvested cells were suspended in Krebs-Ringer Bicarbonate HEPES buffer (KRBH: NaCl 125 mM, CaCl_2_ 1 mM, MgSO_4_ 1.2 mM, KCl 4.74 mM, NaHCO_3_ 5 mM, BSA 0.1%, pH 7.4) with 2.8 or 16.7 mM of glucose at ca. 4 × 10^6^ cells/mL. These glucose concentrations were chosen at the lower and higher ends of the sigmoid response of β-cells to glucose. Cellular respiration was measured and, when required, 1 μg/mL oligomycin was added. The fraction of O_2_ consumption used for producing ATP was calculated as Equation (2).

fraction of O_2_ consumption = (basal − oligomycin)/basal (2)

### 2.6. Other Measurements

To estimate the level of oxidative species present inside cells at the end of the exposure period to cadmium, INS-1 cells were grown and treated with CdCl2 as above (Section 2.1) for 96 h, harvested and rinsed with PBS (1 mL/0.5 × 10^6^ cells). Dihydroethidium (DHE) at the final concentration of 5 μM was added to the cell suspension which was then incubated 30 min in the dark at 37 °C, and cells were analyzed for fluorescence with a LSR Fortessa™ cell analyzer (Becton Dickinson, Le Pont de Claix, France).

To measure the concentration of cadmium inside cells, the latter were harvested, rinsed and dry pellets were kept at −80 °C before measurements. The latter were carried out by Inductively-coupled plasma-mass spectrometry (ICP-MS) as described in details elsewhere [22]. An aliquot of the cell preparation was lysed and the protein concentration was measured with the bicinchoninic acid method (Uptima–Interchim) to calibrate the results.

### 2.7. Statistical Analysis

The implemented statistical tests were adjusted to the design of the different experiments, the nature of the measured parameters, and the kind of comparison to be made. The Kruskal–Wallis test or One Way Analysis of Variance, depending on the data distributions, were applied to the parameters tested to vary with the cadmium exposure group. For microscopic image analysis, the Dunn’s test was used to compare each group to the one which was not exposed to cadmium. The difference of rank means, the Q test statistic, and the *p* < 0.05 status were recorded.

## 3. Results

### 3.1. Effects of Cadmium on INS-1 Cells

#### 3.1.1. Viability of INS-1 Cells upon Long-Term Exposure to Cadmium

**Figure 1:**
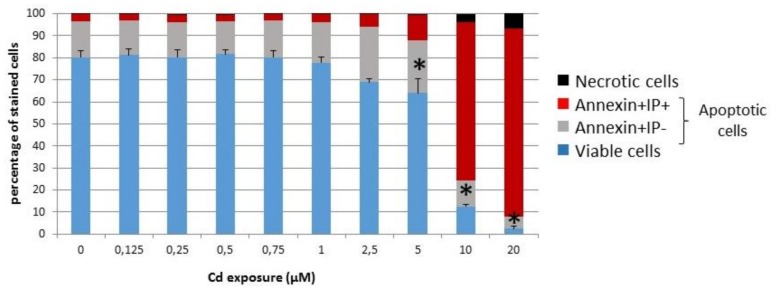
Viability of INS-1 cells in the presence of cadmium. The fractions of viable and death committed cells after 72 h of cadmium exposure were measured by flow cytometry after FluoProbe 488-Annexin V and PI labeling. Only above 2.5 μM was the fraction of viable cells significantly altered. * ANOVA test for live cells *p* < 0.05 vs. 0 μM Cd^2+^ (*n* = 4).

Since the aim of the present study is to probe the sensitivity of the INS-1 cell line upon long-term exposure to sub-lethal concentrations of cadmium, these β-cells were kept for the maximal amount of time in culture in the presence of cadmium before analysis. It was observed that a given batch of cells could not sustain growth for more than ca. 4 days before reaching high density for those not exposed or dying for the most exposed ones, i.e., without applying the stress associated with passage in suspension. Hence, 4 days was the time limit set for the following experiments. The analysis of viability after 72 h indicated that the proportion of viable cells was not significantly altered below 2.5 μM (Figure 1). By further keeping cells for an additional day in the presence of cadmium, the decreasing fraction of viable cells as a function of the cadmium concentration was measured by the ability of cells to reduce a tetrazolium compound. The median lethal dose was calculated at 5.0 μM with a standard error of 1.35 in several experiments.

From the above, it was decided that only cadmium concentrations well below the median lethal dose would be worth considering in an effort to mimic chronic low-level exposure. Therefore, further data were obtained by exposing cells to concentrations up to 2 or 2.5 μM, for 96 and 72 h, respectively, these upper points being taken as limits corresponding to the onset of cell death, of less than 15% in each case.

#### 3.1.2. Cadmium Uptake

**Figure 2:**
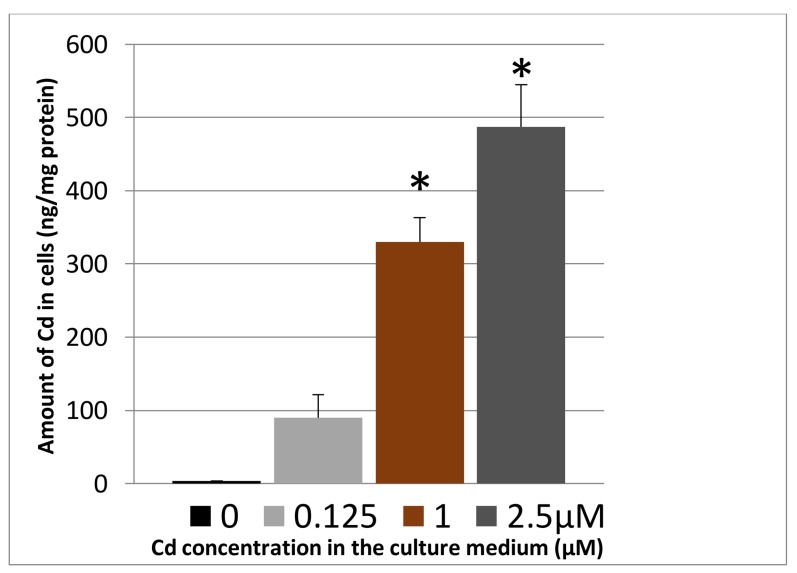
Cadmium uptake by INS-1 cells. The amount of cadmium in washed cells was measured by inductively-coupled plasma-mass spectrometry (ICP-MS) after 72 h of cadmium exposure and normalized to the protein concentration. * ANOVA test, *p* < 0.001 vs. 0 μM Cd^2+^ (*n* = 3).

Despite the sub-lethal cadmium concentrations used in the experimental setup defined in Section 3.1.1, cadmium did enter and accumulated inside cells in a dose-dependent way (Figure 2).

### 3.2. Organization of the Mitochondrial Network and Impact of Long-Term Cadmium Exposure

#### 3.2.1. Microscopic Examination of Mitochondria

**Figure 3:**
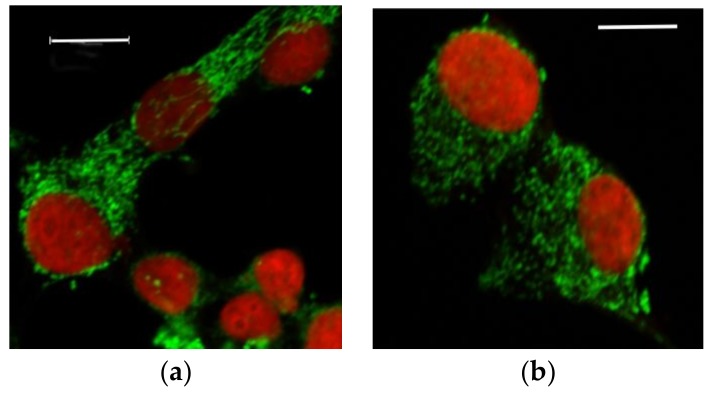
Selected images showing the effect of sub-lethal doses of cadmium on INS-1 cells mitochondria. Mitochondrial morphology was followed by staining a mitochondrial protein of the matrix, aconitase (green). The nucleus was stained by PI (red). INS-1 cells were treated for 96 h with (**a**) 0 and (**b**) 2 μM CdCl_2_. Scale bars 10 μm.

The visualization of mitochondria by immunofluorescence staining showed that the aspect of the mitochondrial network changed after exposure of cells to doses of cadmium for 96 h (Figure 3). The continuous interconnected reticulum seen for non-exposed cells was gradually transformed into a set of discrete and isolated organelles. The strings of mitochondria disappeared fully at the highest cadmium concentration of 2 μM.

#### 3.2.2. Quantitative Analysis of the Mitochondrial Network

**Figure 4:**
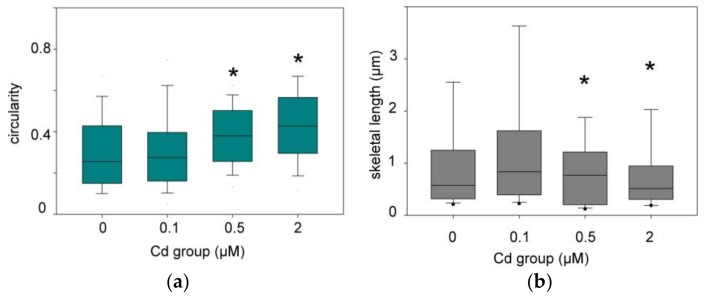
Box Plots showing the distribution of circularity (**a**) and skeletal lengths (**b**) of the mitochondrial network after 96 h of exposure to cadmium. * Kruskal–Wallis test *p* < 0.05 for the indicated groups compared to the reference one (0). The boundaries are the 25th and 75th percentiles and the median and error bars are plotted.

The microscopic images were treated as described in the Material and Methods section. Two parameters, circularity and skeletal length, were selected for analysis, and the results of comparisons between cadmium treatment groups and control INS-1 cells are shown in Figure 4. Exposure of cells to 0.1 μM cadmium did not produce patterns that were significantly different from those of untreated cells. However, at 0.5 μM cadmium and above the shape parameters were different as compared to the non-treated group. It can be noted that the differences in Figure 4 appear more clearly for circularity, which is a relative value involving two parameters derived from similarly treated images (see Section 2.3), than for the skeletal length which is an absolute value that strongly depends on the way the images were filtered.

### 3.3. Functional Analysis of Mitochondria

#### 3.3.1. ATP Production by INS-1 Cells after Long Term Exposure to Moderate Levels of Cadmium

One of the main functions of mitochondria is to convert energy in the form of ATP. It was thus of interest to examine whether the reorganization of the mitochondrial network observed in Section 3.2 translated into any change in the production of ATP.

**Figure 5:**
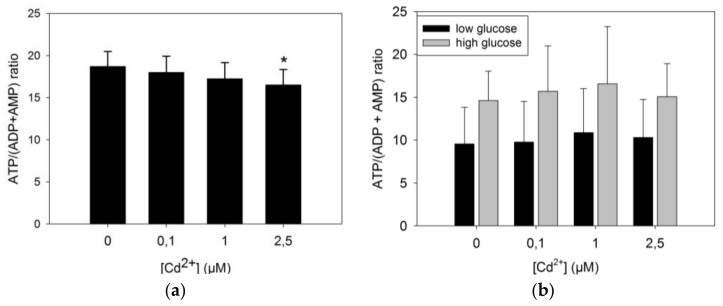
ATP/(ADP + AMP) ratios in INS-1 cells exposed to cadmium. (**a**) INS-1 cells were exposed to the indicated concentrations of cadmium for 72 h in the complete growth medium, and the cellular concentrations of nucleotides were measured as described in Section 2.4. A *t*-test was applied to compare each group to the non-exposed one. * *p* = 0.02, *n* = 9 for each group; (**b**) After the cadmium treatment, cells were pre-incubated for 1 h in KRBH buffer supplemented with 2.8 mM glucose, and then for another hour in the same medium (black bars) and with 16.7 mM glucose (grey bars); *n* = 7 for each group.

The ATP/(ADP + AMP) ratio under standard growth conditions (Figure 5a) was found to decrease in a dose-dependent way, and the difference between the control group of unexposed cells and exposed ones reached statistical significance at 2.5 μM CdCl_2_. Islets of Langerhans produce insulin in response to variations of glucose concentrations, and mitochondria are responsible for increased ATP production under these conditions. The latter was thus measured in response to increased glucose concentrations (Figure 5b). High (16.7 mM) glucose concentrations increased the ATP load of the cells as expected by a factor of approximately 50% on average as compared to low (2.7 mM) glucose concentrations. But when comparing among the cadmium treatment groups, no statistical differences were found between the energy charges of these cells.

#### 3.3.2. Oxygen Consumption of INS-1 Cells after Long Term Exposure to Moderate Levels of Cadmium

**Figure 6:**
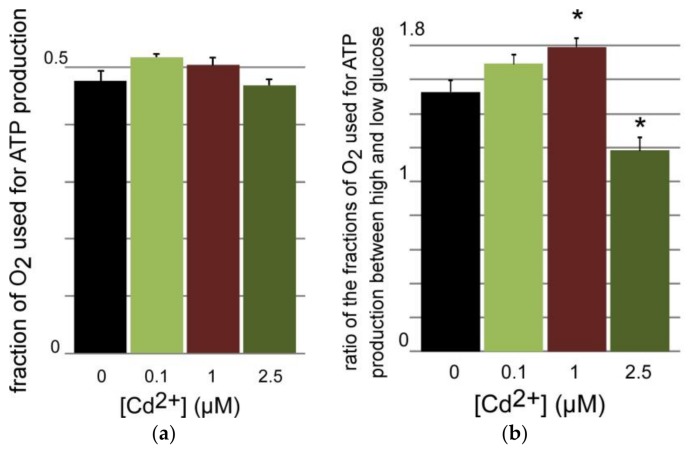
Respiration of INS-1 cells after 72 h-exposure to CdCl_2_. (**a**) Respiration rates were measured as described in Section 2.5 and the slope ratios [(basal – oligomycin)/basal] were calculated for each cadmium treatment group (*n* = 3); (**b**) The same experiment was carried out in KRBH buffer supplemented with either 16.7 or 2.8 mM glucose (*n* = 3–5). The ratio of the values obtained for each series are plotted. * One-way Anova *p* values of 0.013 (1 μM) and 0.002 (2.5 μM) as compared to the control group.

As above for nucleotide measurements, the respiration rates were first recorded for intact cells in the complete growth medium. The basal respiration rate was compared with that of the same cells in the presence of oligomycin that inhibits the proton channel of the F_0_F_1_ complex V. From these data it is possible to calculate the fraction of oxygen consumption used to produce mitochondrial ATP (see Section 2.5). The plot of the results in Figure 6a shows that this fraction did not vary with the cadmium treatment below concentrations triggering cell death. Accordingly, the level of oxidative species within similarly treated cells did not change as a function of the cadmium dose as determined by reaction with dihydroethidium (see Section 2.6). The same procedure was applied to cells that were incubated in serum-free medium with either low (2.8 mM) or high (16.7 mM) glucose. As expected, respiration rates with high glucose concentrations were higher than with low concentrations, and the use of oligomycin enabled to determine which fraction of O_2_ consumption was used for ATP synthesis. The ratio of these fractions directed to ATP under high glucose and low glucose was calculated at each cadmium concentration (Figure 6b). This ratio, which is a proxy of the coupling between glucose increase and ATP production, was found to increase in a dose-dependent way up to 1 μM, but it decreased significantly at 2.5 μM when compared to the other groups.

## 4. Discussion

Considering all the data together, it appears that some characteristics of the INS-1 cell line are sensitive to sub-lethal cadmium concentrations for several generations largely before any sign of significant cellular death is detected. Under the implemented conditions, whereas the decrease of viability becomes significant only largely above 1 μM (Figure 1), the amount of cadmium accumulated over 3 days exceeds the control value by an approximate factor of 300 already at this concentration, and this factor further increases at higher concentrations (Figure 2). β-cells are very specialized in that they almost exclusively convert glucose input above the basal cellular needs into insulin production and secretion [16]. Hence mitochondria represent a first sub-cellular hub downstream of glucose absorption to generate potassium channel inhibition at the plasma membrane via increased ATP.

The prolonged exposure to sub-lethal concentrations of cadmium and the associated increase of accumulated cadmium can be correlated with a reorganization of the mitochondrial network (Figure 3 and Figure 4). The change of mitochondrial morphology is characterized by increasingly fragmented and perinuclear mitochondria when the cadmium dose increases. Since the size of the individual mitochondria does not seem to notably change with application of cadmium, at the level of resolution afforded by the method we implemented to detect them, it may be proposed that the mitochondrial fusion-fission equilibrium is disturbed in favor of fission. Fragmented mitochondria have often been associated with impaired function [23], including in β-cells [24]. They are also observed in β-cells of Type 2 diabetic patients [25], together with altered amounts of selected mitochondrial proteins, decreased energy charge, and depolarized mitochondrial membranes. Post-fission isolated mitochondria may have a variable polarization status, but those that cannot repolarized are directed to mitophagy and loss of function [26]. Thus, the change of mitochondrial morphology that was clearly detected by the circularity parameter at the chronic concentration of 0.5 μM CdCl_2_ and above (Figure 4) might have been expected to lead to impaired ability of the organelles to produce ATP in response to increased glucose.

But the apparent change of mitochondrial fusion-fission dynamics is differentially paralleled by functional consequences. The decreasing trend of the energetic charge, estimated by the ATP/(ADP + AMP) ratio as a function of cadmium exposure, is statistically significant only at the onset of cell death, i.e., at a 3 day-dose of 2.5 μM (Figure 5a). This result indicates that the rate of ATP production may decrease at relatively high cadmium doses, or its use is increased to counter the effects of the cadmium burden, or both. The decreasing trend of Figure 5a is observed when cells are steadily growing in the conventional growth medium containing ca. 11 mM (2 g/L) glucose. Under these conditions the O_2_ respiration rates devoted to ATP production do not change (Figure 6a). This suggests that the decrease of the energetic charge observed in Figure 5a is due to increased ATP consumption or a small decoupling of the mitochondria in the 2.5 μM Cd-group. The relatively high respiration flow that is not used for ATP synthesis (ca. 50% in Figure 6a) is noteworthy, and it has already been noticed in β-cells models [27].

But, when glucose is decreased to 2.8 mM in minimal medium, the energy charge decreases and the difference between the 2.5 μM Cd-group and non-exposed cells cancels out. Similarly, the response to increased glucose does not change for all cadmium-treatment groups (Figure 5b). However, the stability of the energy charge among the different cadmium groups contrasts with the O_2_ consumption devoted to ATP production which first increases as a function of cadmium exposure to reach significance at 1 μM, and then partly collapses at 2.5 μM (Figure 6b), i.e., at the onset of cell death. This phenomenon indicates that challenged cells increase the electron flow to maintain an adjusted response to increasing glucose at sub-lethal cadmium doses, but that cadmium concentrations such as 2.5 μM triggering cell death change this response and no longer request reallocation of the fraction of O_2_ consumption used to produce ATP (Figure 6b) to respond to increasing glucose (Figure 5b).

Changes of mitochondrial morphology and function in β-cells have already been correlated with apoptosis [28,29]. But changes in morphology are not straightforwardly related to changes in glucose-stimulated insulin secretion [30]. Since β-cells can be replenished, the functional consequences of their apoptosis should occur when the β-cells mass is decreased together with insulin production [31]. The dynamics of β-cells turnover endow these cells with an efficient compensatory mechanism in line with loss of β-cells mass being a relatively late development in diabetes, of Type 2 in particular. In a parallel way in the present study, no significant effects on the monitored parameters were observed by applying 0.1 μM cadmium for 3 or 4 days. Only a slight tendency was sometimes noticed (energetic charge, O_2_ consumption upon glucose challenge) that eventually could become significant at higher cadmium concentrations. In this respect, mitochondrial dysfunction cannot be considered as an early sign of cadmium poisoning of β-cells, and any biomarker related to mitochondrial integrity is unlikely to provide a reliable and sensitive probe of mild exposure to cadmium.

These data are another illustration of one of the ways cells try to adapt to an environmental stress [32]. It is likely that the implemented molecular changes triggered by the increasing cadmium load (Figure 2) are numerous, but they do not translate into modified phenotypic traits up to relatively high concentrations (≥0.5 μM). This can be compared to the situation encountered with populations exposed to environmental cadmium. They usually do not exhibit early health problems, but their chronic exposure is expected to impact molecular networks [5,33] hence contributing to morbidity and association with the development of various diseases [4].

But long-life provision of cadmium to pancreatic β-cells is likely to eventually lead to dysfunction, including of their mitochondria. The data reported herein apply to a simple cell model that has been exposed for a relatively short time as compared to the conditions that apply to long-lived animals, humans in particular. This is an obvious and important limitation of this work. However, the data suggest that non-invasive detection of cadmium in islets of Langerhans may provide a useful marker for subjects at risk of developing diabetes. Methods to detect cadmium in vivo are developing [34], and they may eventually become instrumental to probe the highly variable cadmium content of human islets [11], to be correlated with the likeliness of developing diabetes. Future work should analyze insulin secretion under the presently implemented conditions, but this single endpoint depends on additional steps lying downstream of mitochondria in the currently accepted mechanism of glucose-stimulated insulin secretion. They include plasma membrane depolarization, calcium signaling, and granules loading for instance, all events that may be sensitive to cadmium exposure, even at low concentrations. Surface receptors may also be sensitive to external cadmium and modulate the β-cells’ response to increased glucose concentrations. In addition, since Cd accumulates, other intra-cellular targets may influence the overall function of these cells. Also, in line with the results reported here, the sensitivity of the mitochondrial fusion-fission proteins to the presence of cadmium should be probed. Available data indicate that a 2-way relationship between mitochondrial network dynamics and sensitivity to cadmium should be expected [35,36,37] along previously delineated mechanisms [5]. Furthermore, the consequences of low-level exposure of mammals to cadmium should be analyzed in details for glucose homeostasis beyond available data. This has been underway with a rat model which demonstrated that females and neonates of these laboratory animals are at risk of pre-diabetic symptoms under different modalities of chronic low level exposure to cadmium [22,38].
